# Antitumor Activity of a *Bupleurum chinense*-Derived Vesicle-like Particle Preparation in MOC1 Oral Squamous Cell Carcinoma Models

**DOI:** 10.3390/biomedicines14102380

**Published:** 2026-10-19

**Authors:** Shihan Li, Guanmeng Zhang, Lijun Zhao, Shuang Wang, Wanzhu Feng, Xinyi Ran, Wenshu Ge

**Affiliations:** Department of General Dentistry II, Peking University School and Hospital of Stomatology, National Center for Stomatology, National Clinical Research Center for Oral Diseases, National Engineering Research Center of Oral Biomaterials and Digital Medical Devices, Beijing 100081, China

**Keywords:** *Bupleurum chinense*, vesicle-like particle preparation, oral squamous cell carcinoma, MOC1, apoptosis, mitochondrial membrane potential, reactive oxygen species

## Abstract

**Background/Objectives:** This study evaluated the antitumor activity of a *Bupleurum chinense*-derived extracellular vesicle-like particle (BC-EVLP) preparation in MOC1 oral squamous cell carcinoma (OSCC) models. **Methods**: Fresh roots were processed by differential centrifugation, filtration, ultracentrifugation, and sucrose density-gradient separation. The preparation was characterized by transmission electron microscopy, nanoparticle tracking analysis, and total protein measurement. Cellular association, viability, ROS-associated fluorescence, mitochondrial membrane potential, and Annexin V-FITC/PI staining were assessed. Cell viability was also compared after exposure to equal added volumes of the preparation or pre-gradient ultracentrifugation supernatant. Female C57BL/6 mice bearing syngeneic MOC1 tumors received peritumoral treatment every 3 days for 14 days, followed by tumor histology. **Results**: The preparation contained nanoscale vesicle-like structures, with median and mean particle diameters of 162.0 and 191.9 nm. DiI-associated fluorescence was detected in MOC1 cells after 4 h. Treatment reduced cell viability and was associated with increased DCF fluorescence, a lower JC-1 red/green ratio, and increased Annexin V-positive fractions. The BC-EVLP preparation reduced cell viability more than the supernatant at equal added volumes, although the higher-volume supernatant treatment retained partial activity. High-dose treatment attenuated tumor growth in vivo, and representative tumor sections showed less densely cellular areas. **Conclusions**: The BC-EVLP preparation exhibited antitumor activity in the tested MOC1 models. The active constituents and the relative contributions of vesicular and non-vesicular components remain unresolved.

## 1. Introduction

Oral cancer remains a global health burden [1], with oral squamous cell carcinoma (OSCC) accounting for most malignancies of the oral cavity [2]. Treatment includes surgery, radiotherapy, and systemic therapy, selected according to disease stage and patient characteristics [3,4]. Immune checkpoint inhibitors have expanded treatment options for patients with head and neck squamous cell carcinoma (HNSCC), including OSCC [5]. More recently, the phase 3 KEYNOTE-689 trial demonstrated that adding perioperative pembrolizumab to standard care improved event-free survival in patients with resectable, locally advanced HNSCC [6]. However, treatment resistance and treatment-related toxicities remain important limitations, and responses to immunotherapy vary among patients [3,4,5]. Modulation of extracellular vesicle secretion by cannabidiol has also been proposed as an investigational strategy, although direct evidence in OSCC remains limited [7]. These unmet needs motivate continued preclinical investigation of additional antitumor candidates, including naturally derived materials whose biological activity and safety require experimental evaluation.

Plant-derived extracellular vesicle-like particles (EVLPs) have attracted increasing attention because they contain diverse lipids, proteins, nucleic acids, and small-molecule constituents and can exert biological effects without exogenous drug loading [8,9,10,11]. Previous studies have reported direct anticancer activity of vesicular preparations derived from several plant species [12,13,14,15,16]. Lemon-derived vesicles promoted oxidative changes and apoptosis in gastric cancer cells [17], while grape callus-derived exosome-like nanoparticles reduced cancer-cell viability and induced apoptosis [18]. Of particular relevance to OSCC, bitter-melon-derived extracellular vesicles inhibited tumor-cell proliferation and enhanced the activity of 5-fluorouracil [19], and *Panax notoginseng*-derived nanovesicles also showed inhibitory effects on OSCC cells [20]. These findings suggest that plant-derived vesicular preparations may represent a source-dependent class of naturally bioactive antitumor particles.

*Bupleurum chinense* DC. is a medicinal plant used in traditional East Asian medicine and contains bioactive constituents, including saikosaponins [21,22]. Several constituents have antiproliferative and pro-apoptotic effects in experimental cancer models [23,24,25]. We therefore investigated a *B. chinense*-derived extracellular vesicle-like particle (BC-EVLP) preparation against OSCC. The term BC-EVLPs is used operationally to denote the isolated particle preparation.

In this study, we characterized the BC-EVLP preparation and evaluated its association with MOC1 cells and its effects on cell viability, Annexin V-FITC/PI staining, intracellular ROS-associated fluorescence, and mitochondrial membrane potential. We also compared its effects on cell viability with those of corresponding supernatants and examined local administration in a syngeneic MOC1 tumor model.

## 2. Materials and Methods

### 2.1. Plant Material

Fresh roots of *Bupleurum chinense* DC. were obtained from a commercial supplier and originated from Dingxi, Gansu Province, China. The material was supplied and labeled as *B. chinense* DC. Upon receipt, the roots were thoroughly washed with sterile water to remove surface contaminants, surface-dried, and immediately processed for BC-EVLP isolation.

### 2.2. Isolation and Enrichment of the BC-EVLP Preparation

BC-EVLPs were prepared by differential centrifugation, ultracentrifugation, and sucrose density-gradient separation. Fresh *B. chinense* roots (250 g) were homogenized with 500 mL of sterile phosphate-buffered saline (PBS) under chilled conditions. The homogenate was passed through four layers of sterile gauze to remove coarse plant fibers and tissue debris.

The filtrate underwent successive centrifugation at 1000× *g* for 10 min, 2000× *g* for 20 min, and 10,000× *g* for 40 min at 4 °C. After each centrifugation step, the supernatant was retained for the subsequent purification procedure. The final supernatant was filtered successively through 0.45 μm and 0.22 μm membranes before ultracentrifugation at 100,000× *g* for 60 min at 4 °C. The supernatant was retained for the comparison described in Section 2.6, and the pellet was resuspended in sterile PBS.

The resuspended pellet was further fractionated by discontinuous sucrose density-gradient ultracentrifugation. Sucrose solutions of 8%, 30%, 45%, and 60% (*w*/*v*) were prepared in PBS, and 8 mL of each solution was sequentially layered in 38.5-mL ultracentrifuge tubes. Approximately 4 mL of the crude BC-EVLP suspension was loaded onto the top of the gradient and centrifuged at 100,000× *g* for 2 h at 4 °C using an SW 32 Ti swinging-bucket rotor (Beckman Coulter Life Sciences, Indianapolis, IN, USA). Approximately 0.5 mL of the particle-enriched fraction at the 30%/45% sucrose interface was collected, diluted with sterile PBS, and ultracentrifuged again at 100,000× *g* for 60 min at 4 °C to remove residual sucrose. The final pellet was resuspended in 1 mL sterile PBS, aliquoted, and stored at −80 °C without repeated freeze–thaw cycles.

### 2.3. Characterization of BC-EVLPs

The particle size distribution and concentration of BC-EVLPs were measured by nanoparticle tracking analysis (NTA) using a ZetaView instrument (Particle Metrix, Inning am Ammersee, Germany) equipped with a 520-nm laser. Samples were diluted 500-fold in PBS and analyzed at approximately 27.6 °C using ZetaView software (version 8.05.14 SP7). Each measurement consisted of one cycle covering 11 positions. Particle concentrations were corrected for the dilution factor and expressed as particles/mL.

Morphological features of BC-EVLPs were visualized by transmission electron microscopy (TEM). A 10 μL aliquot of the particle suspension was placed on a copper grid and retained for 2 min at room temperature. After removal of excess liquid, the grid was negatively stained with 2% (*w*/*v*) phosphotungstic acid for 3 min. Following air drying for 10 min at room temperature, samples were imaged using a JEM-1400PLUS transmission electron microscope (JEOL Ltd., Akishima, Tokyo, Japan).

The protein concentration of the BC-EVLP preparation was determined using a BCA Protein Assay Kit (Servicebio, Wuhan, China) with bovine serum albumin (BSA) as the standard, according to the manufacturer’s instructions. The BC-EVLP sample was diluted 10-fold in PBS before measurement to ensure that the absorbance value fell within the range of the standard curve. Absorbance was measured at 562 nm, and the protein concentration was calculated from the BSA standard curve and corrected for the dilution factor. Particle yield was calculated by normalizing the total particle number recovered in the final BC-EVLP suspension to the initial fresh weight of *B. chinense* roots and was expressed as particles/g fresh tissue. The particle-to-protein ratio was calculated from the NTA particle concentration and BCA protein concentration and expressed as particles/μg protein.

### 2.4. Cell Culture

The murine OSCC cell line MOC1 was kindly provided by Yuguang Wang’s research group at Peking University School of Stomatology (Beijing, China). Cells were cultured in Dulbecco’s modified Eagle medium (DMEM; Gibco, Grand Island, NY, USA) supplemented with 10% fetal bovine serum (FBS; Gibco, Grand Island, NY, USA) and 1% penicillin–streptomycin at 37 °C in a humidified atmosphere containing 5% CO_2_. Cells were passaged at approximately 70–80% confluence, and those in the logarithmic growth phase were used for subsequent experiments.

### 2.5. Cellular Uptake of BC-EVLPs

BC-EVLPs were fluorescently labeled with DiI (Beyotime Biotechnology, Shanghai, China) according to the manufacturer’s protocol. Free dye was removed by ultracentrifugation at 100,000× *g* for 60 min, after which the particle pellet was resuspended in sterile PBS.

MOC1 cells grown in confocal imaging dishes were exposed to DiI-labeled BC-EVLPs at 1 × 10^8^ particles/mL for 4 h at 37 °C. After washing with PBS, the cells were fixed and permeabilized, followed by staining of the actin cytoskeleton with Actin-Tracker Green (Beyotime Biotechnology, Shanghai, China) and nuclear counterstaining with Hoechst (Beyotime Biotechnology, Shanghai, China). Cellular uptake was examined by confocal laser scanning microscopy.

### 2.6. CCK-8 Cell Viability Assay

Cell viability after BC-EVLP exposure was quantified with a Cell Counting Kit-8 (CCK-8; Beyotime Biotechnology, Shanghai, China). MOC1 cells were plated in 96-well plates at 5 × 10^3^ cells/well and allowed to attach overnight. The following day, cells were exposed to BC-EVLPs at 1 × 10^6^–1 × 10^10^ particles/mL for 24 h. CCK-8 reagent (10 μL) was then added to each well, followed by incubation at 37 °C for 1 h. Absorbance at 450 nm was recorded with a microplate reader, and cell viability was expressed relative to untreated cells. In a separate comparison, MOC1 cells were exposed for 24 h to BC-EVLP preparations at 1 × 10^9^ or 1 × 10^10^ particles/mL or to supernatant collected after the first ultracentrifugation of the filtered root homogenate at 100,000× *g* for 60 min at 4 °C, before sucrose-gradient separation. Sup-L and Sup-H denote supernatant treatments added at the same volumes as the final BC-EVLP stock used for the low- and high-concentration treatments, respectively.

### 2.7. Live/Dead Cell Staining

MOC1 cell survival after BC-EVLP exposure was further examined by Calcein-AM/PI staining. Cells were exposed to BC-EVLPs at 1 × 10^9^ and 1 × 10^10^ particles/mL for 24 h, rinsed with PBS, and processed with a Calcein/PI Cell Viability/Cytotoxicity Assay Kit (Beyotime Biotechnology, Shanghai, China) following the manufacturer’s protocol. Fluorescence images were obtained with a BX53 microscope (Olympus Corporation, Hachioji, Tokyo, Japan) using the same acquisition settings for all groups.

### 2.8. Intracellular ROS Measurement

Intracellular ROS-associated fluorescence was detected with DCFH-DA. MOC1 cells were exposed to BC-EVLPs at 1 × 10^9^ and 1 × 10^10^ particles/mL for 24 h, followed by incubation with DCFH-DA working solution at 37 °C in the dark in accordance with the manufacturer’s protocol. After PBS washing, cells were harvested and DCF fluorescence was evaluated by flow cytometry [26,27]. Untreated cells were used as the control.

### 2.9. Mitochondrial Membrane Potential Measurement

Mitochondrial membrane potential was examined using a JC-1 assay kit (Servicebio, Wuhan, China). MOC1 cells were exposed to BC-EVLPs at 1 × 10^9^ and 1 × 10^10^ particles/mL for 24 h. After removal of the culture medium, cells were rinsed with PBS and incubated with JC-1 working solution at 37 °C in the dark for 20 min according to the manufacturer’s protocol. The cells were then washed with the supplied assay buffer, and JC-1 aggregate-associated red fluorescence and monomer-associated green fluorescence were visualized with a BX53 fluorescence microscope (Olympus Corporation, Hachioji, Tokyo, Japan). Changes in the red/green fluorescence pattern were used to evaluate mitochondrial membrane potential, with a shift toward green fluorescence indicating membrane depolarization [28].

### 2.10. Apoptosis Analysis

Apoptotic cells were quantified by Annexin V-FITC/PI staining. MOC1 cells were exposed to BC-EVLPs at 1 × 10^9^ and 1 × 10^10^ particles/mL for 48 h. To avoid loss of detached apoptotic cells, both floating and adherent fractions were collected. After PBS washing, the cells were resuspended in 1× Annexin V Binding Buffer and labeled with a FITC Annexin V Apoptosis Detection Kit (BD Biosciences, San Jose, CA, USA) according to the manufacturer’s protocol.

Samples were acquired on a NovoCyte flow cytometer (Agilent Technologies, Santa Clara, CA, USA). Cell debris was excluded based on forward- and side-scatter characteristics, and single cells were selected before Annexin V-FITC/PI analysis. Fluorescence compensation between the FITC and PI channels was performed using appropriate single-stained controls. A minimum of 10,000 events were acquired for each sample. Cells were classified as Annexin V-negative/PI-negative, Annexin V-positive/PI-negative, Annexin V-positive/PI-positive, or Annexin V-negative/PI-positive. The combined Annexin V-positive fraction was used as an operational apoptosis-associated endpoint. The double-positive population includes late apoptotic or otherwise membrane-compromised cells and does not independently establish the mode of cell death [29]. Data were obtained from three independent experiments and analyzed using a consistent gating strategy.

### 2.11. MOC1 Syngeneic Tumor Model and BC-EVLP Treatment

Animal procedures were approved by the Experimental Animal Ethics Committee of Peking University School and Hospital of Stomatology (approval no. BDKQ-202507160754) and were conducted in compliance with applicable animal welfare regulations. Reporting followed the ARRIVE guidelines [30]. Six-week-old female C57BL/6 mice were maintained under specific pathogen-free conditions. Syngeneic tumors were established by subcutaneous inoculation of 5 × 10^6^ MOC1 cells per mouse.

When tumors reached approximately 5 mm in diameter, mice were randomized into three groups (*n* = 6 per group): PBS control, low-dose BC-EVLP, and high-dose BC-EVLP. BC-EVLPs were delivered peritumorally at 1 × 10^9^ or 1 × 10^10^ particles/mL, respectively, quantities of 200 μL per mouse. These concentrations corresponded to 2 × 10^8^ and 2 × 10^9^ particles per dose, respectively. Injections were given every 3 days for 14 days, while control animals received 200 μL PBS by the same route.

Tumor dimensions were measured using a digital caliper on days 0, 2, 5, 8, 11, and 14 after treatment initiation. Tumor volume was calculated as V = L × W^2^/2, where L and W represent the longest and shortest tumor diameters, respectively, with L ≥ W. Body weight was recorded throughout treatment. At the study endpoint, mice were deeply anesthetized and euthanized by cervical dislocation, and tumors were excised for gross imaging and histological examination. Total body weight includes tumor mass and was not considered sufficient evidence of systemic safety.

For histological examination, tumor tissues from all six mice per group were fixed in 4% paraformaldehyde, paraffin-embedded, and sectioned at a thickness of 5 μm. Sections were deparaffinized, rehydrated, and stained with hematoxylin and eosin (H&E) according to standard histological procedures. Representative fields were selected from H&E-stained tumor sections for qualitative histopathological assessment and imaged using an Olympus BX53 light microscope (Olympus Corporation, Hachioji, Tokyo, Japan).

### 2.12. Statistical Analysis

Data are presented as mean ± SD unless otherwise specified. Statistical analyses were performed using GraphPad Prism 10 (GraphPad Software, Boston, MA, USA). Comparisons among three or more independent groups were performed using one-way ANOVA followed by Tukey’s multiple-comparisons test, as appropriate. Apoptosis data from three independent experiments were analyzed using ordinary one-way ANOVA followed by Tukey’s multiple-comparisons test. The supernatant comparison was analyzed using one-way repeated-measures ANOVA with the Geisser–Greenhouse correction followed by Tukey’s multiple-comparisons test. Longitudinal tumor-volume data were analyzed using two-way repeated-measures ANOVA with the Geisser–Greenhouse correction, followed by Dunnett’s multiple-comparisons test for comparisons of each BC-EVLP treatment group with the PBS control at individual time points. Sample sizes are indicated in the figure legends, and technical replicates were not considered independent biological observations. A *p* value < 0.05 was considered statistically significant.

## 3. Results

### 3.1. Physicochemical Characterization and Cellular Uptake of BC-EVLPs

**Figure 1:**
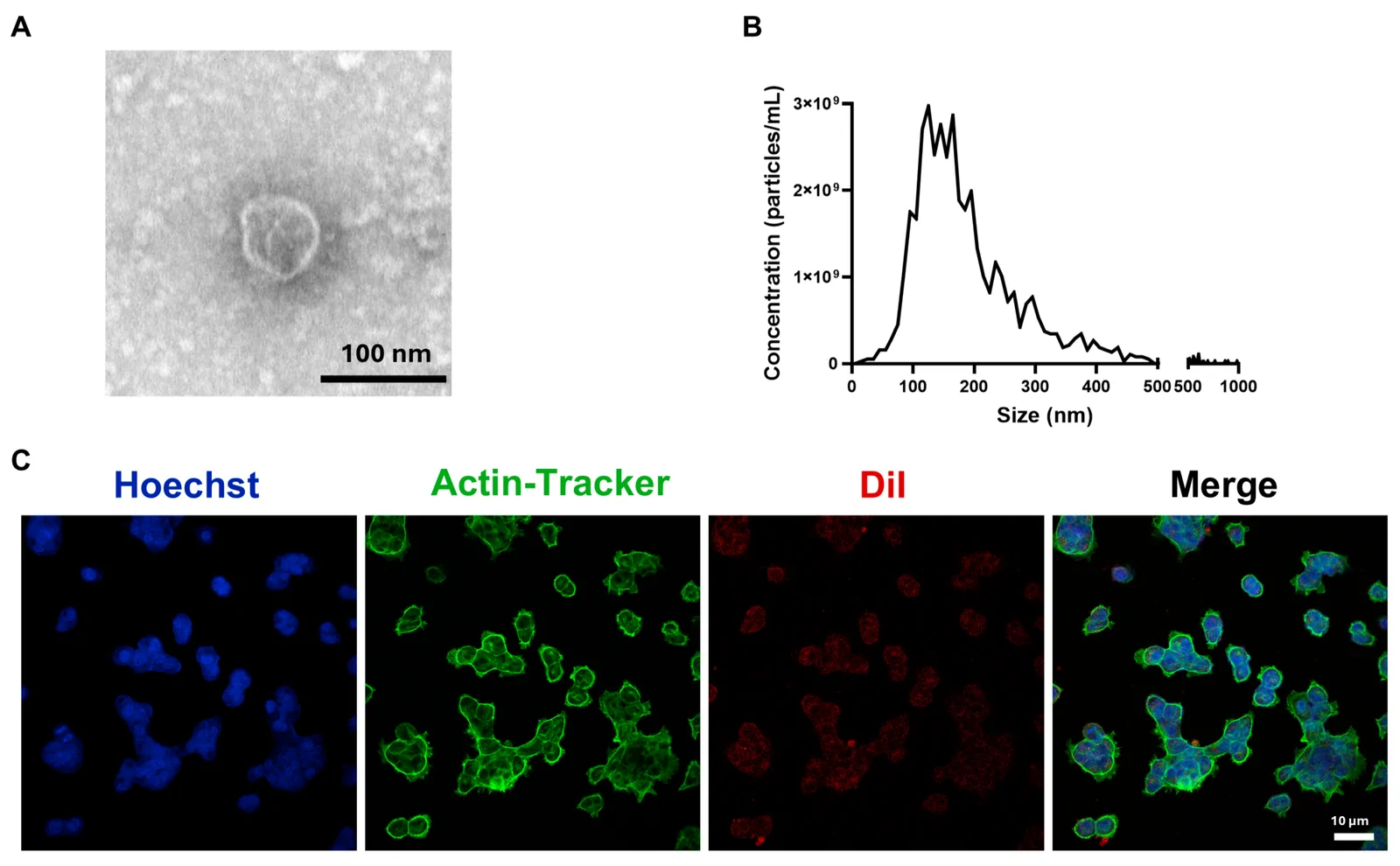
Physicochemical characterization and cellular uptake of BC-EVLPs. (**A**) Representative TEM image. (**B**) NTA particle size distribution. (**C**) Confocal images of MOC1 cells incubated with DiI-labeled BC-EVLPs at 1 × 10^8^ particles/mL for 4 h. Red: DiI; green: Actin-Tracker Green; blue: Hoechst. Scale bars: 100 nm (**A**) and 10 μm (**C**).

TEM revealed rounded or cup-shaped vesicle-like structures in the BC-EVLP preparation (Figure 1A). NTA demonstrated a predominantly nanoscale particle distribution, with the major population concentrated at approximately 100–300 nm. The median particle diameter was 162.0 nm, the mean diameter was 191.9 nm, and the particle concentration was 4.1 × 10^10^ particles/mL (Figure 1B). The total protein concentration of the BC-EVLP preparation, determined by BCA assay using a BSA standard curve (Figure S1A), was 1.4 mg/mL (1400 μg/mL). Based on the corresponding particle concentration, the particle-to-protein ratio was approximately 2.9 × 10^7^ particles/μg protein. With a final resuspension volume of 1 mL obtained from 250 g of fresh *B. chinense* roots, the particle yield was approximately 1.64 × 10^8^ particles/g fresh tissue.

After 4 h of incubation with MOC1 cells, the DiI-labeled preparation produced red fluorescent signals predominantly in cytoplasmic and perinuclear regions (Figure 1C). These observations are consistent with cellular association or uptake of the labeled preparation.

### 3.2. BC-EVLPs Reduce MOC1 Cell Viability

**Figure 2:**
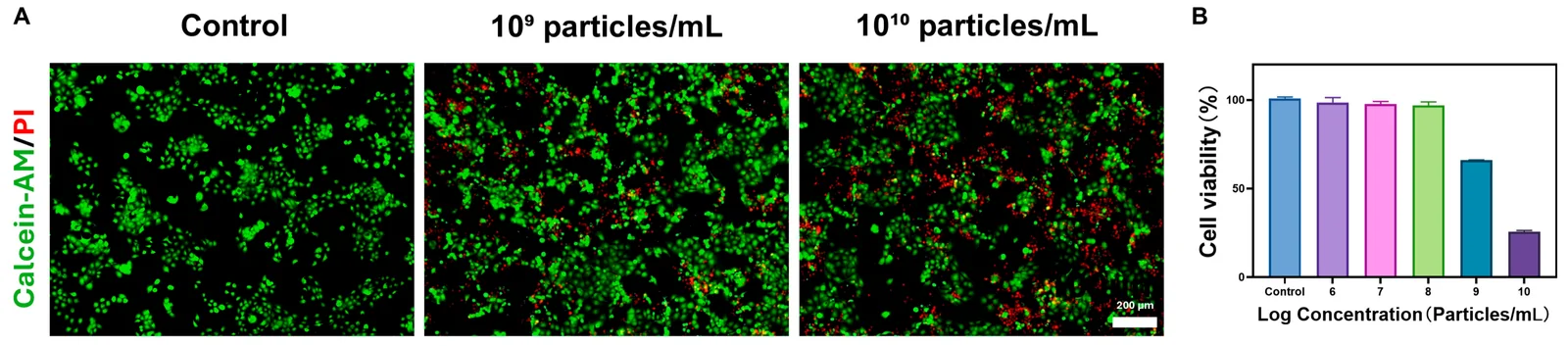
Effects of BC-EVLPs on MOC1 cell viability after 24 h of treatment. (**A**) Representative Calcein-AM/PI images of untreated cells and cells treated with 1 × 10^9^ or 1 × 10^10^ particles/mL. Green: viable cells; red: membrane-compromised cells. Scale bar: 200 μm. (**B**) CCK-8 cell viability at 1 × 10^6^–1 × 10^10^ particles/mL, normalized to the untreated control. Axis values 6–10 denote log_10_ particle concentration. Data are presented as mean ± SD (*n* = 3).

Calcein-AM/PI staining revealed more red fluorescent membrane-compromised cells after 24 h of BC-EVLP treatment at 1 × 10^9^ and 1 × 10^10^ particles/mL than in the untreated control (Figure 2A). CCK-8 analysis demonstrated a concentration-related reduction in MOC1 cell viability, which was most pronounced at 1 × 10^9^ and 1 × 10^10^ particles/mL after 24 h of treatment (Figure 2B). In the additional comparison, BC-EVLP preparations reduced cell viability more than the pre-gradient ultracentrifugation supernatant at equal added volumes (Figure S1B). The higher-volume supernatant treatment (Sup-H) also showed partial inhibitory activity.

### 3.3. BC-EVLP Treatment Is Associated with Increased Intracellular ROS and Reduced Mitochondrial Membrane Potential

**Figure 3:**
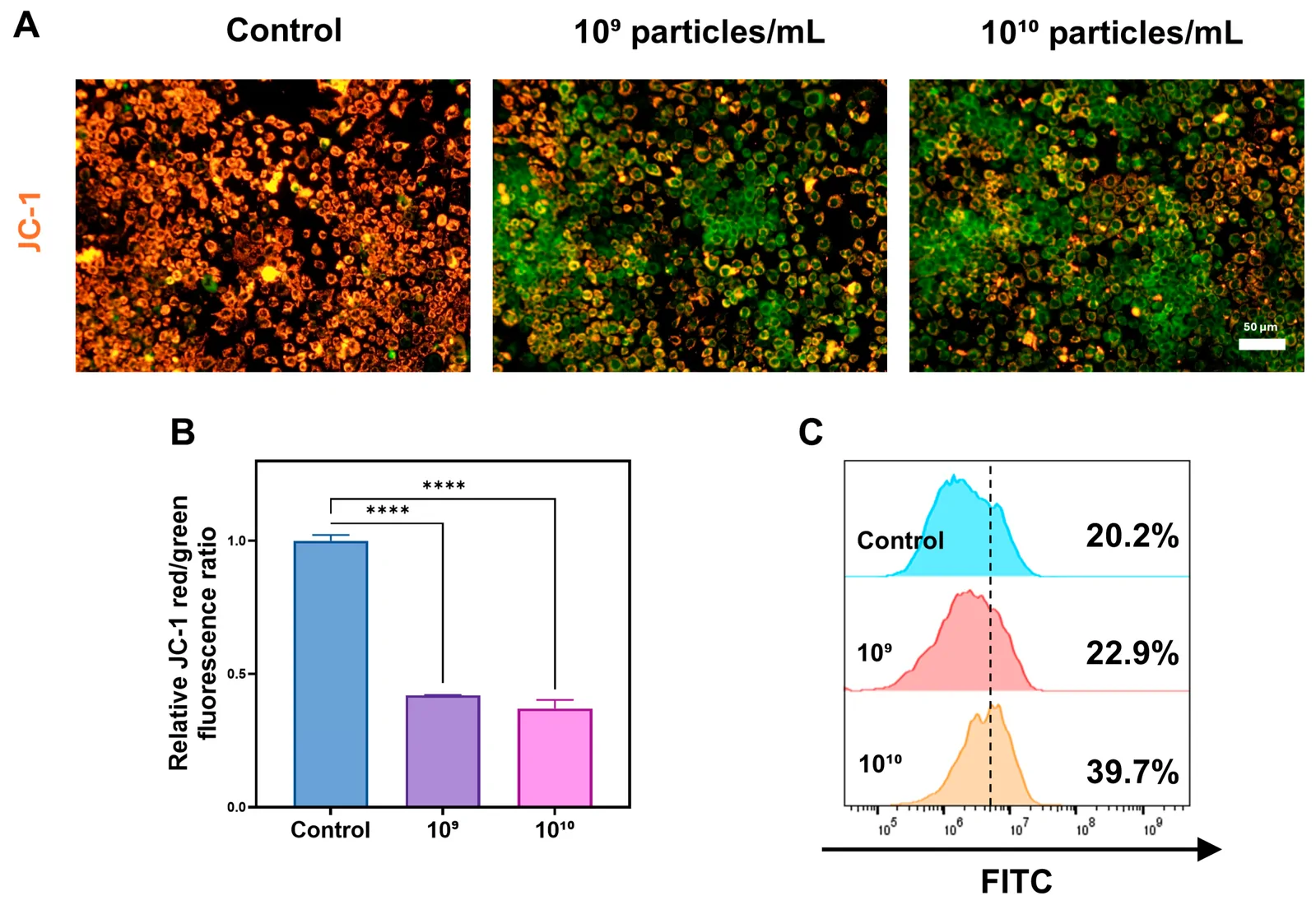
Effects of BC-EVLPs on mitochondrial membrane potential and intracellular ROS-associated fluorescence in MOC1 cells after 24 h. Cells were treated with 1 × 10^9^ or 1 × 10^10^ particles/mL. (**A**) Representative JC-1 fluorescence images. Red: JC-1 aggregates, indicating relatively high mitochondrial membrane potential; green: JC-1 monomers, associated with reduced mitochondrial membrane potential. Scale bar: 50 μm. (**B**) JC-1 red/green fluorescence ratio. Data are mean ± SD (*n* = 3); one-way ANOVA with Tukey’s multiple-comparisons test, **** *p* < 0.0001. (**C**) Representative DCF flow-cytometry distributions; percentages indicate events above the dashed threshold.

JC-1 staining showed reduced red aggregate fluorescence relative to green monomer fluorescence after 24 h of BC-EVLP treatment (Figure 3A). The red/green fluorescence ratio was lower in both treatment groups than in the control, consistent with mitochondrial membrane depolarization (Figure 3B). DCF flow-cytometry profiles showed a rightward shift in fluorescence distribution, which was more pronounced at 1 × 10^10^ particles/mL (Figure 3C). Together, these findings indicate that BC-EVLP treatment was associated with increased ROS-associated fluorescence and reduced mitochondrial membrane potential.

### 3.4. Annexin V-FITC/PI Staining After BC-EVLP Treatment

**Figure 4:**
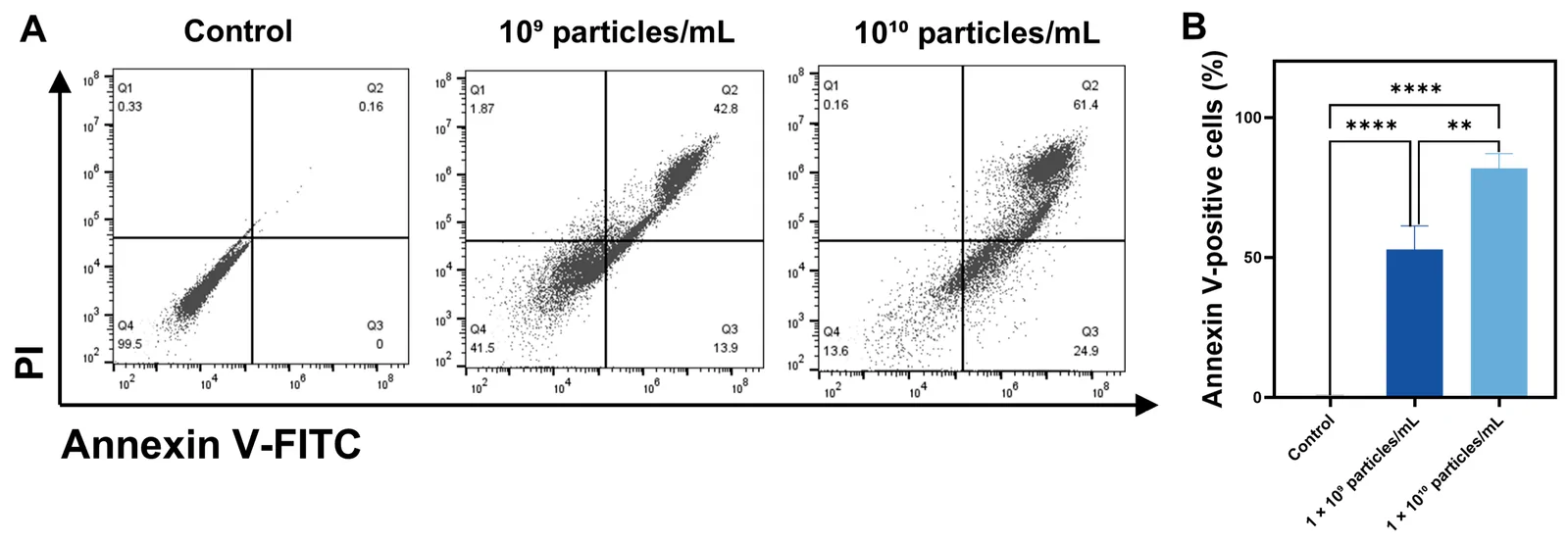
Annexin V-FITC/PI staining in MOC1 cells after 48 h of BC-EVLP treatment. (**A**) Representative Annexin V-FITC/PI flow-cytometry plots of untreated MOC1 cells and cells treated with BC-EVLPs at 1 × 10^9^ or 1 × 10^10^ particles/mL for 48 h. Cells were classified according to Annexin V-FITC and PI staining. (**B**) Quantification of the combined Annexin V-positive fraction, calculated as the sum of Annexin V^+^/PI^−^ and Annexin V^+^/PI^+^ populations. Data are presented as mean ± SD from three independent experiments (*n* = 3). Statistical analysis was performed using ordinary one-way ANOVA followed by Tukey’s multiple-comparisons test. ** *p* < 0.01, **** *p* < 0.0001 for the bracketed comparisons.

Representative Annexin V-FITC/PI plots showed increased Annexin V-positive populations after 48 h of BC-EVLP treatment (Figure 4A). In the displayed examples, the combined Annexin V-positive fractions were approximately 56.7% and 86.3% at 1 × 10^9^ and 1 × 10^10^ particles/mL, respectively. Quantification across three independent experiments showed higher combined Annexin V-positive fractions in both treatment groups than in the control, with a further increase at the higher concentration (Figure 4B).

### 3.5. Evaluation in the MOC1 Syngeneic Tumor Model

**Figure 5:**
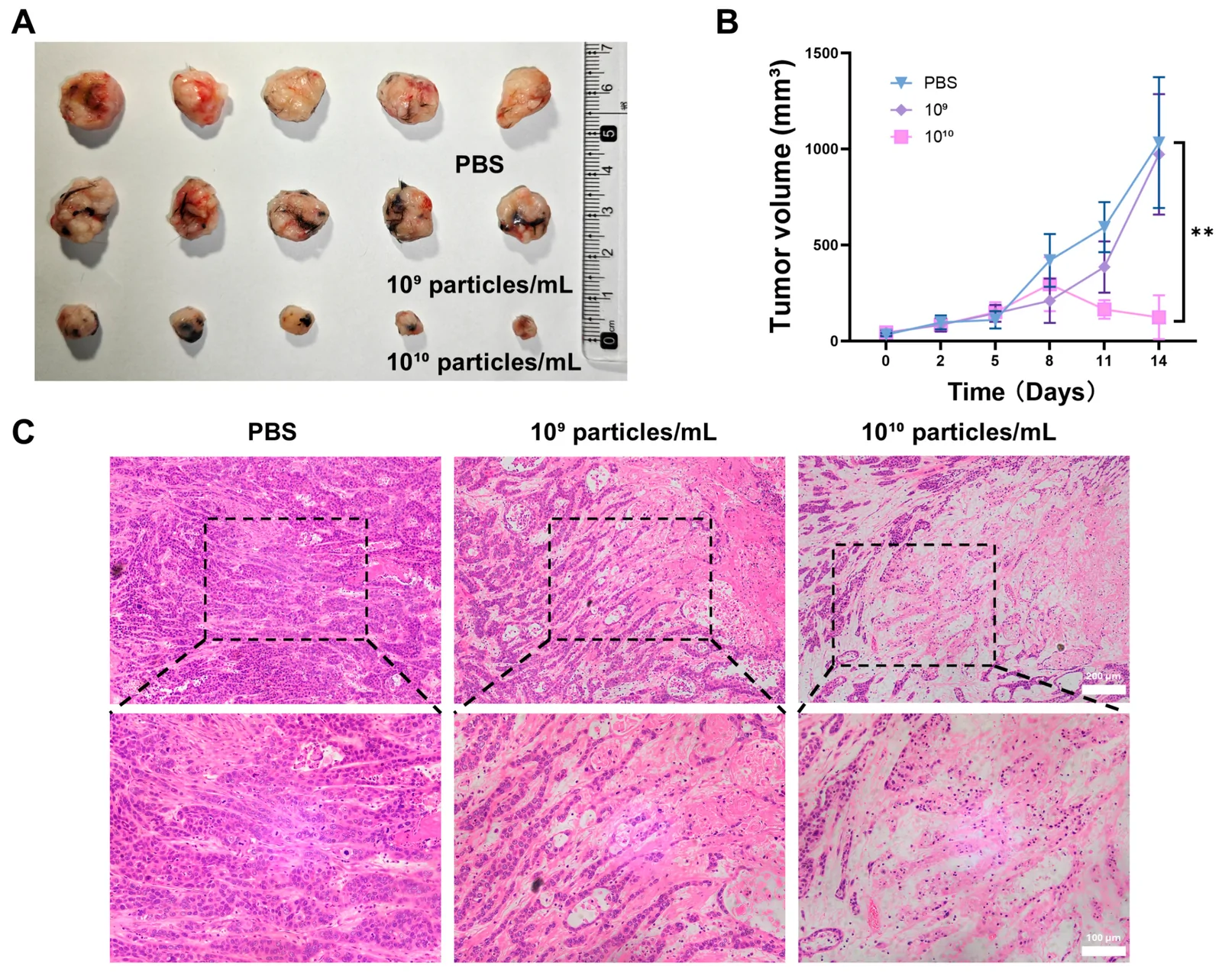
Evaluation of peritumoral BC-EVLP administration in female C57BL/6 mice bearing MOC1 syngeneic tumors. (**A**) Representative photographs of excised tumors from the PBS control, low-dose, and high-dose groups. Five tumors are displayed per group for consistent presentation because one tumor was damaged during excision; all six mice per group were retained in the tumor-growth analysis. (**B**) Tumor growth curves during the 14-day treatment period. Tumor volume was calculated as V = L × W^2^/2, where L and W represent the longest and shortest tumor diameters, respectively. Data are presented as mean ± SD (*n* = 6 mice per group). Longitudinal tumor-volume data were analyzed using two-way repeated-measures ANOVA with the Geisser–Greenhouse correction, followed by Dunnett’s multiple-comparisons test for comparisons of each BC-EVLP treatment group with the PBS control at individual time points. For visual clarity, only the day 14 comparison between the PBS and high-dose BC-EVLP groups is indicated in the figure. ** *p* < 0.01 for the indicated comparison (adjusted *p* = 0.0014). (**C**) Representative H&E-stained tumor sections from six mice per group. Higher-magnification views of the boxed regions are shown below; scale bars are indicated in the images. BC-EVLPs were administered peritumorally at 2 × 10^8^ or 2 × 10^9^ particles per injection in 200 μL, corresponding to the 10^9^ and 10^10^ particles/mL labels, respectively; control mice received 200 μL PBS.

Gross photographs of excised tumors are shown in Figure 5A. Longitudinal analysis demonstrated a treatment-dependent difference in tumor growth (Figure 5B). Compared with the PBS control, the low-dose BC-EVLP group showed significantly lower tumor volumes on days 8 and 11, but this difference was not maintained on day 14. In contrast, the high-dose BC-EVLP group showed significantly lower tumor volumes on days 11 and 14, indicating a more sustained inhibitory effect at the higher dose. In representative H&E-stained sections, PBS-treated tumors contained densely cellular regions, whereas high-dose BC-EVLP-treated tumors showed more extensive eosinophilic, hypocellular areas (Figure 5C). These histopathological observations were qualitative and were not subjected to morphometric quantification. Total body-weight changes are presented in Figure S2. Because total body weight includes tumor mass, these measurements alone were not considered sufficient evidence of systemic safety.

## 4. Discussion

The BC-EVLP preparation reduced MOC1 cell viability and was associated with increased intracellular DCF fluorescence, a lower JC-1 red/green ratio, and increased Annexin V-positive fractions. High-dose peritumoral administration also attenuated tumor growth, accompanied by qualitative histological differences in the examined tumor sections. The preparation produced a greater reduction in cell viability than the pre-gradient ultracentrifugation supernatant at equal added volumes, although activity remained in Sup-H. These findings extend the investigation of plant-derived particle preparations in experimental cancer models [12,13,14,15,16,31].

The present findings are consistent with evidence that plant-derived vesicle-like particles can exert direct anticancer effects. Lemon-derived extracellular vesicles induced ROS accumulation and apoptosis in gastric cancer cells [17], while bitter-melon-derived extracellular vesicles inhibited OSCC proliferation and enhanced 5-fluorouracil activity [19]. Grape callus-derived nanoparticles reduced cancer-cell viability and promoted apoptosis and cell-cycle arrest [18]. Apoptosis-associated effects have been reported for cucumber- and fingerroot-derived particles [32,33], while selected plant sap-derived preparations reduced tumor-cell viability [34], and citrus-derived nanovesicles suppressed leukemia xenograft growth [35]. Ginseng- and Artemisia-derived preparations also inhibited tumor growth through effects on tumor-associated macrophages [36,37]. A study comparing vesicle-like preparations from *Panax notoginseng* tubers of different sizes further suggests that source-material characteristics may influence their composition and antitumor activity [20]. Antitumor activity without exogenous drug loading has therefore been reported as a source-dependent property of plant-derived vesicular preparations [15,16,38,39,40].

BC-EVLP treatment was accompanied by increased intracellular DCF fluorescence, reduced mitochondrial membrane potential, and increased Annexin V-positive fractions. These changes are consistent with oxidative and mitochondrial perturbation associated with apoptotic cell death [27,28,29], as reported for other plant-derived preparations [17,19,32,33]. DCF fluorescence does not identify a specific ROS species, and the present data do not establish ROS as an upstream mediator of apoptosis [26,27,29]. Antioxidant rescue or mitochondria-targeted interventions are needed to clarify this relationship.

The active constituents of the BC-EVLP preparation remain unidentified. Plant-derived vesicular preparations can contain biologically active lipids, proteins, nucleic acids, and secondary metabolites [11,39,41], but the reported antitumor properties of saikosaponins and other *B. chinense* constituents do not establish their presence or localization in this preparation [21,22,23,24,25]. The preparation reduced cell viability more than the pre-gradient ultracentrifugation supernatant at equal added volumes, although Sup-H retained partial activity. These fractions were not normalized to the same amount of starting plant material, and residual particles in the supernatant were not quantified. The comparison therefore does not resolve the contributions of soluble constituents, particle-associated cargo, or co-sedimenting material, or establish whether intact vesicles are required. BCA measurements and particle-to-protein ratios characterize the preparation without establishing chemical purity; chemical profiling and further fraction-based functional studies are needed to identify the active components and their association with the particles [40,41,42].

The use of homogenized roots also limits conclusions about particle origin. TEM and NTA characterize morphology and size but cannot establish extracellular biogenesis or exclude intracellular membrane fragments [42,43,44]. Isolation from apoplastic washing fluids samples a different compartment from tissue homogenization [45,46]. Accordingly, BC-EVLPs is an operational term for the enriched preparation rather than a designation of confirmed extracellular or endosomal origin [47,48]. DiI-associated cellular fluorescence likewise supports association or uptake of labeled material without identifying the internalized components.

The findings are limited to MOC1 cells and a subcutaneous syngeneic tumor model in female C57BL/6 mice receiving peritumoral injections. Additional OSCC models, normal oral epithelial cells, and alternative administration routes are needed to assess generalizability and tumor selectivity. Tumor histology was evaluated qualitatively and does not establish a specific cell-death mechanism. Biodistribution, pharmacokinetics, and comprehensive systemic safety remain to be evaluated [38,39,49,50,51,52]; total body weight alone is insufficient to establish safety because it includes tumor mass.

## 5. Conclusions

The BC-EVLP preparation reduced MOC1 cell viability and was associated with oxidative and mitochondrial changes and increased Annexin V-positive fractions. High-dose local administration attenuated tumor growth in the syngeneic MOC1 model. These findings support further preclinical investigation, with the active constituents and the relative contributions of vesicular and non-vesicular components still to be defined.

## Data Availability

The raw data supporting the conclusions of this article will be made available by the authors on request.

## Supplementary Materials

The following supporting information can be downloaded at https://www.mdpi.com/article/10.3390/biomedicines14102380/s1, Figure S1: Protein quantification and comparison of the BC-EVLP preparation with pre-gradient ultracentrifugation supernatant; Figure S2: Total body-weight changes during the 14-day treatment period in mice receiving PBS or peritumoral BC-EVLPs. The 10^9^ and 10^10^ labels denote formulation concentrations in particles/mL, corresponding to 2 × 10^8^ and 2 × 10^9^ particles per injection in 200 μL. Data are presented as mean ± SD (n = 6 mice per group). Body weight includes tumor mass and was not interpreted as a stand-alone measure of systemic safety.

## Abbreviations

The following abbreviations are used in this manuscript:

BC-EVLPs	*Bupleurum chinense*-derived extracellular vesicle-like particles
CCK-8	Cell Counting Kit-8
DCF	2′,7′-dichlorofluorescein
DMEM	Dulbecco’s modified Eagle medium
FBS	fetal bovine serum
NTA	nanoparticle tracking analysis
OSCC	oral squamous cell carcinoma
PBS	phosphate-buffered saline
PI	propidium iodide
ROS	reactive oxygen species
TEM	transmission electron microscopy
