# Supplementary material for: Antitumor Activity of a Bupleurum chinense-Derived Vesicle-like Particle Preparation in MOC1 Oral Squamous Cell Carcinoma Models
Source: Biomedicines. 2026 Oct 19;14(10):2380. doi: 10.3390/biomedicines14102380 (PMC13701900; doi:10.3390/biomedicines14102380)
Supplement: Supplementary file 1 [file biomedicines-14-02380-s001.zip › biomedicines-4604150-supplementary.pdf]

# Supplementary Materials

## Antitumor Activity of a *Bupleurum chinense*-Derived Vesicle-like Particle Preparation in MOC1 Oral Squamous Cell Carcinoma Models

**Shihan Li, Guanmeng Zhang, Lijun Zhao, Shuang Wang, Wanzhu Feng, Xinyi Ran and Wenshu Ge ***

Department of General Dentistry II, Peking University School and Hospital of Stomatology, National Center for Stomatology, National Clinical Research Center for Oral Diseases, National Engineering Research Center of Oral Biomaterials and Digital Medical Devices, Beijing 100081, China

*  Correspondence: esther1234@hsc.pku.edu.cn (W.G.)

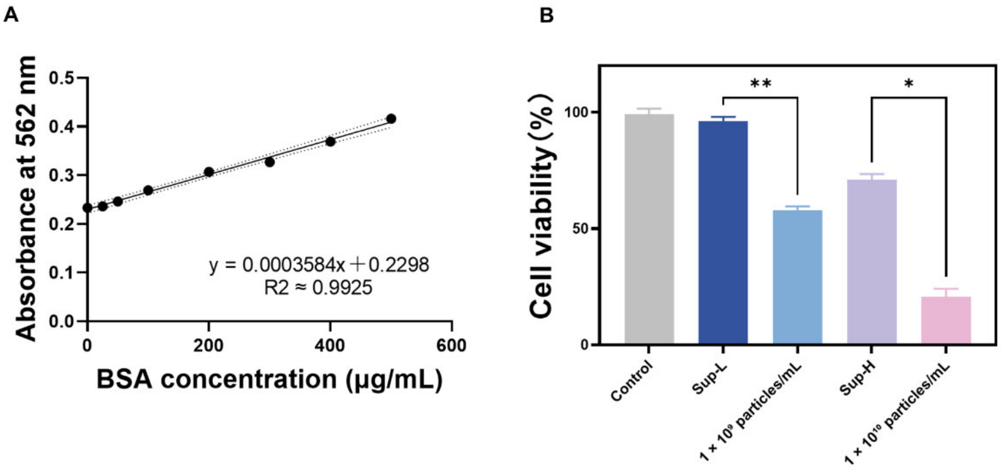


Figure S1. Protein quantification and comparison of the BC-EVLP preparation with pre-gradient ultracentrifugation supernatant.

(A) Bovine serum albumin (BSA) standard curve for the bicinchoninic acid (BCA) protein assay, measured at 562 nm. (B) MOC1 cell viability after 24 h of treatment with BC-EVLP preparations at $1 \times 10^9$ or $1 \times 10^{10}$ particles/mL or supernatant collected after the first ultracentrifugation of the filtered root homogenate at 100,000 × g for 60 min at 4 °C, before sucrose-gradient separation. Sup-L and Sup-H denote supernatant treatments added at the same volumes as the final BC-EVLP stock used for the low- and high-concentration treatments, respectively; the fractions were not normalized to the same amount of starting plant material. Residual particle concentrations in the supernatant were not measured by NTA. Cell viability was normalized to the untreated control. Data are presented as mean ± SD (n = 3). Statistical comparisons were performed using one-way repeated-measures ANOVA with the Geisser–Greenhouse correction followed by Tukey's multiple-comparisons test. * $p < 0.05$ and ** $p < 0.01$ for the bracketed comparisons.

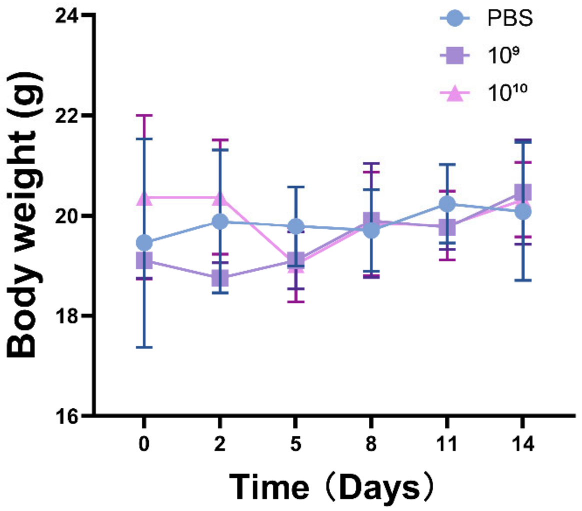


Figure S2. Total body-weight changes during the 14-day treatment period in mice receiving PBS or peritumoral BC-EVLPs. The $10^9$ and $10^{10}$ labels denote formulation concentrations in particles/mL, corresponding to $2 \times 10^8$ and $2 \times 10^9$ particles per injection in 200 μL. Data are presented as mean ± SD (n = 6 mice per group). Body weight includes tumor mass and was not interpreted as a stand-alone measure of systemic safety.
